# Supplementary material for: Spatial fine-mapping for gene-by-environment effects identifies risk hot spots for schizophrenia
Source: Nat Commun. 2018 Dec 13;9:5296. doi: 10.1038/s41467-018-07708-7 (PMC6294340; doi:10.1038/s41467-018-07708-7)
Supplement: Supplementary file 1 — Supplementary Information [file 41467_2018_7708_MOESM1_ESM.pdf]

Spatial fine-mapping for gene-by-environment effects identifies risk hot spots for schizophrenia.

Fan et al.

A Supplementary Methods

A.1 Deriving the Voronoi tessellations through adaptive binning

To ensure each region has a approximately the same number of individuals while maintaining spatial resolution for risk mapping, we define environs based on a adaptive Voronoi tessellation process that been used in the field of astronomy and computer vision. Given a point pattern $\{p_1, ..., p_n\}$ on $S \subset R^2$, the Voronoi tessellation divides S into n distinct cells $\{C_i, ..., C_n\}$, such that each cell C_i contains only locations closer to their corresponding point p_i than all other point $p_{j \neq i}$. Hence, the tessellation makes the area of a given environs inversely proportional to the population density in that region. However, given the 1 km^2 resolution of the original spatial data, the capital area can have 154 individuals in the same cube while many rural area have at most 1 observation. To account for the vast differences across regions, we merged each observation with their immediate neighbors to ensure the number of individuals in each tessellation has similar amount of individuals. To maintain the property of Voronoi tessellation, the neighbors were based on the Delaunay triangulation, the dual of the Voronoi tessellation. This is inherently a spatially adaptive process because the Delaunay neighbors are defined by the relative distance comparing to all other points, not by absolute distance between points. The final results are illustrated in Supplementary Figure 1. As Supplementary Figure 1 a shown, the variations on the number of individuals in each tessellation are constrained within 60 to 197 with median in 105. The variations are independent of the size of the locale, as intended. Supplementary Figure 1 b-c illustrate the Delaunay-Voronoi duality that is the basis for the locale definition. Importantly, the final results are based on entire cohort members, enhance no sampling uncertainties for tessellation process.

A.2 Simulations for estimating procedures

To examine whether E and GxE components can be captured by our method, and also the impact of weak genetic instrument, we performed a Monte Carlo simulations based on our model estimates. While keeping the sample sizes and numbers of tessellations fixed, we vary different noise levels in the PRS to see how it deviates the estimation away from the true values of GxE effects. The results from Supplementary Figure 3 were based on 1000 iterations of simulations. As noted in the simulation, the accuracy of polygenic risk score (PRS) is proportional to how much the GxE variations can be estimated. Therefore, PRS as a weak proxy would reduce the statistical power of our analysis. Despite of this caveat, the GxE effects were still significantly associated with risk of schizophrenia.

A.3 Multilevel model for spatial related risk of schizophrenia

In the context of survival analysis for case-cohort samples[24,25], the multilevel mixed effects model can be represented as the following,

$$\begin{aligned}
\lambda(t|G_j, i) &= \lambda_0(t) \exp(u_i^E + X^T\beta + G_j^T(\beta + \gamma_i^{G\times E})) \\
u_i^E &\sim N(0, \sigma_E^2) \\
\gamma_i^{G\times E} &\sim N(0, \sigma_{G\times E}^2)
\end{aligned}$$

where λ is the hazard function, t is the time-to-event for jth subject has a hospitalization with discharge code corresponding to schizophrenia (ICD10 - F20), u_i^E and $\gamma_i^{G\times E}$ are random intercept and random slope at locale i, and G_j is the PRS for the jth subject. Covariates X include age when registry information was collected (December 31, 2013), gender, family history, population density of each tessellation, and first three genetic principal components of genetic ancestry. We weight the likelihood of each observation using its inverse sampling probability as the case-cohort design, thereby giving unbiased parameter estimates for the country of Denmark as a whole. Because the cox proportional hazard model canceled out the baseline hazard terms during the fitting, the model does not contain the individual's residual error term. The random components here represent the variations due to the risk differences between locales. In this model formulation, the E term represent the locale of upbringing effects independent from genetic effects (G). The gene-by-environment (GxE) term captures modulation of locale of upbringing given an individuals genetic liability for schizophrenia.

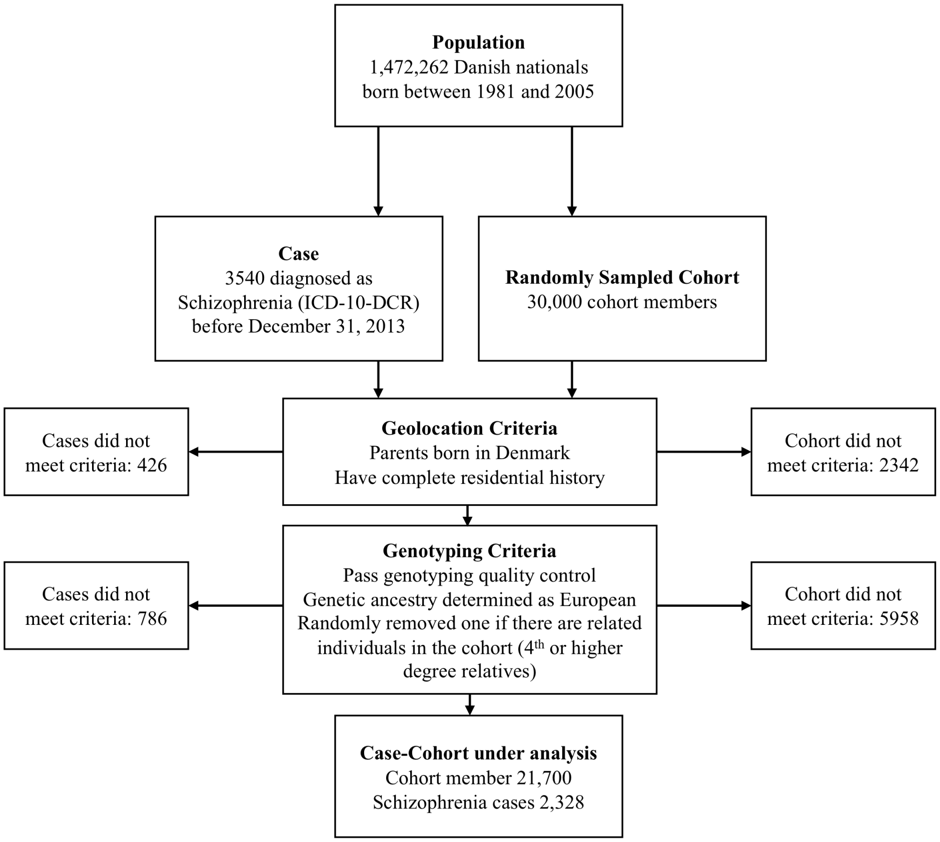

Supplementary Figure 1: The flow chart of the samples involved in current analysis.

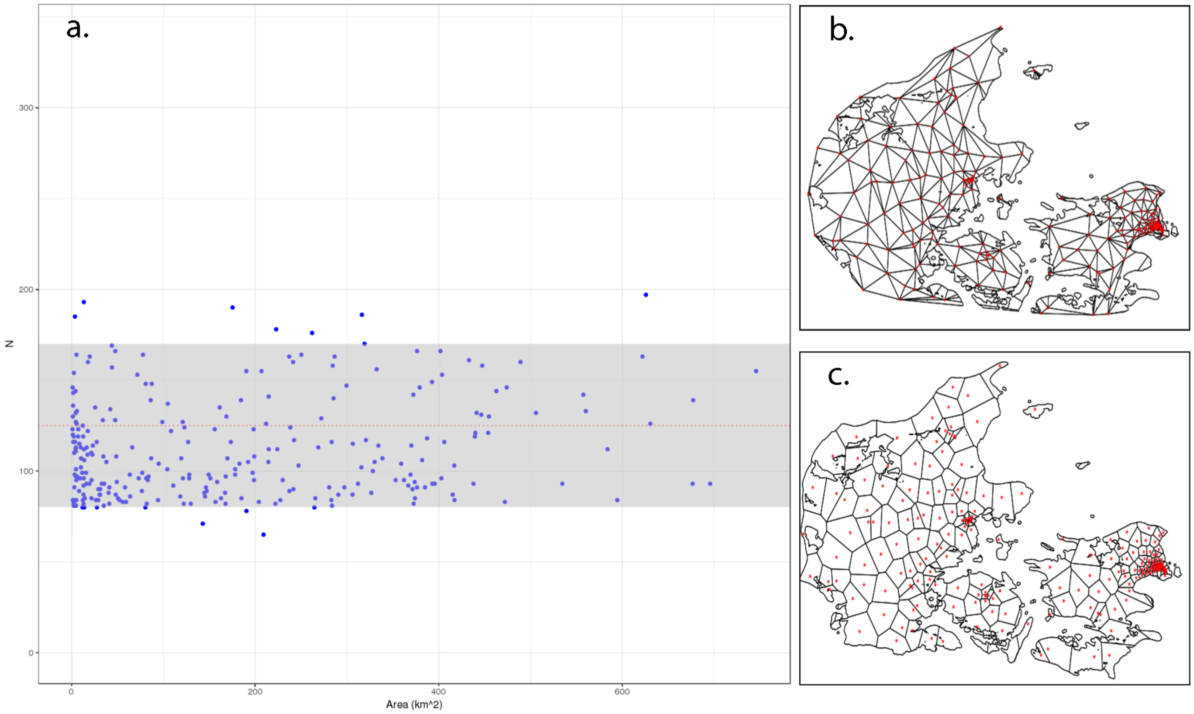

Supplementary Figure 2: Illustration of tessellation process. Each observation was merged with its neighbors to make the number of subjects in a given tessellation fall into the gray shaded region. a. The resulting binned observations. Each blue dot represent one tessellation. The variations are independent of the size of the tessellation. b. Delaunay triangulation between each binned observation. Delaunay triangulation linked neighbors by comparing with all other points in the 2D map. Therefore, the number of neighbors are not biased toward urban region. Red dots are each binned centroid. c. Voronoi tessellation based on the final delaunay triangulation. This formed the basis for the locale definition.

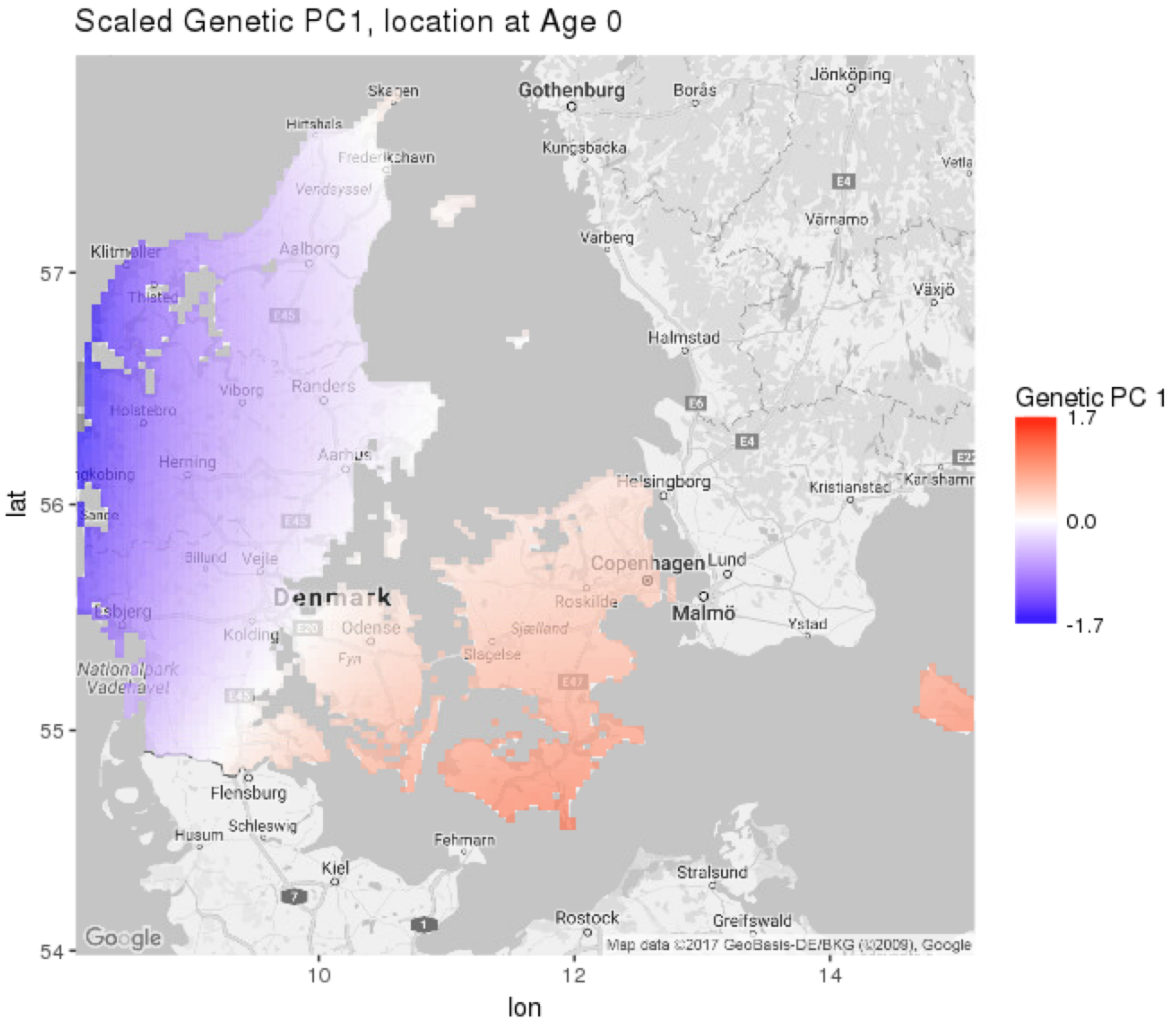

Supplementary Figure 3: Clines of the first genetic principal component. The smoothing clines are based on the predicted values of genetic PC1 from the third order polynomials on longitude and lattitudes. All coefficients for the cubic fit on longitude and lattitude have p values less than $1e^{-16}$ except the third order of the longitude. The model indicates an apparent northwesting to southeasting cline.

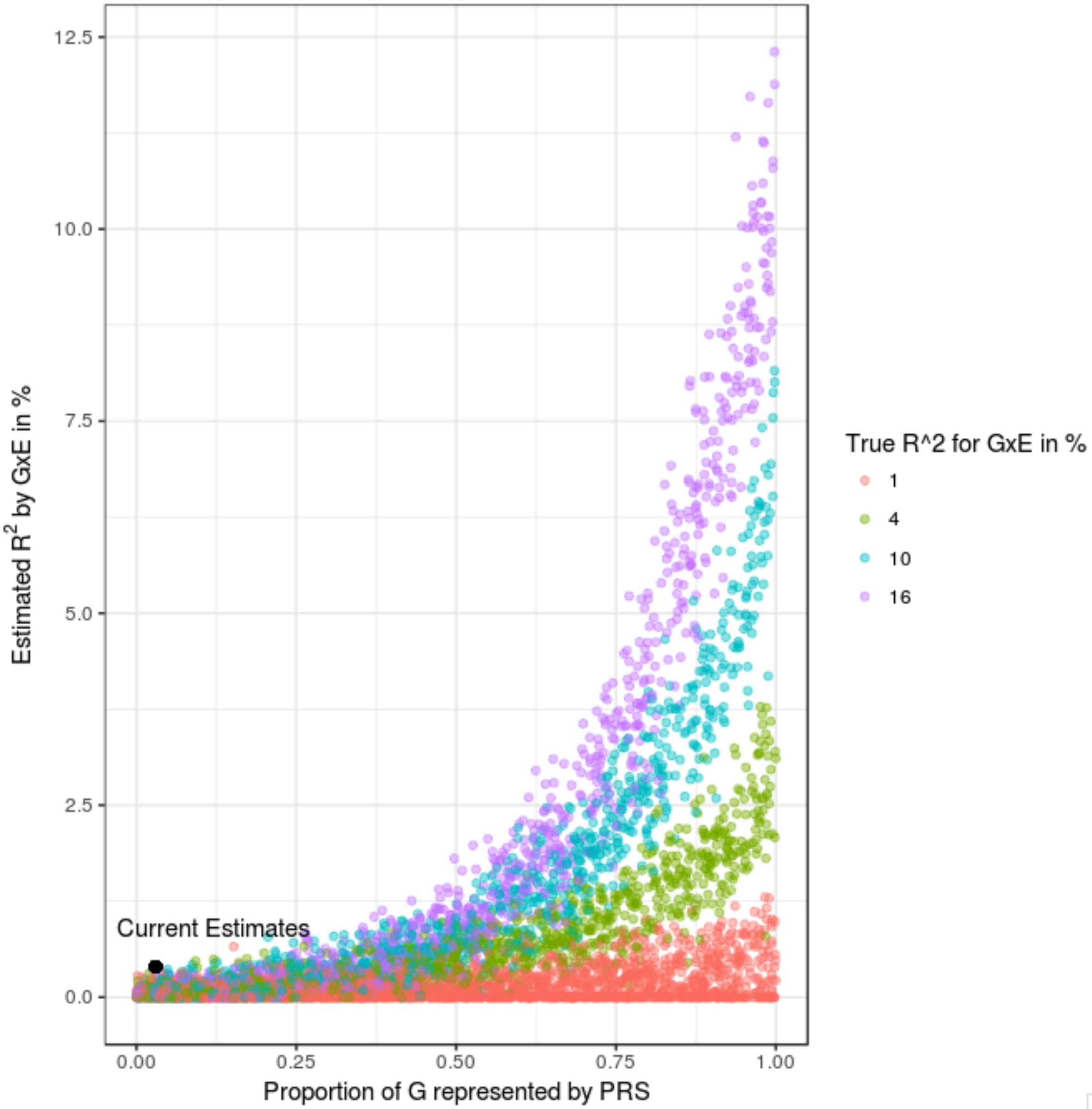

Supplementary Figure 4: C Sensitivity analyses for the impact of missing genetic risks on the GxE effects. Each colored dots represent one simulation result, 1000 in total. Four simulated true GxE values were used here, 1%, 4%, 10%, and 16% while the proportion of genetic effects characterized by PRS varied from almost 0 to 100%. The estimate based on log-normal model for current case-cohort data has variance explained R2 as 05% with PRS explaining less than 3% of SNP heritability, specified in the plot with color in black.

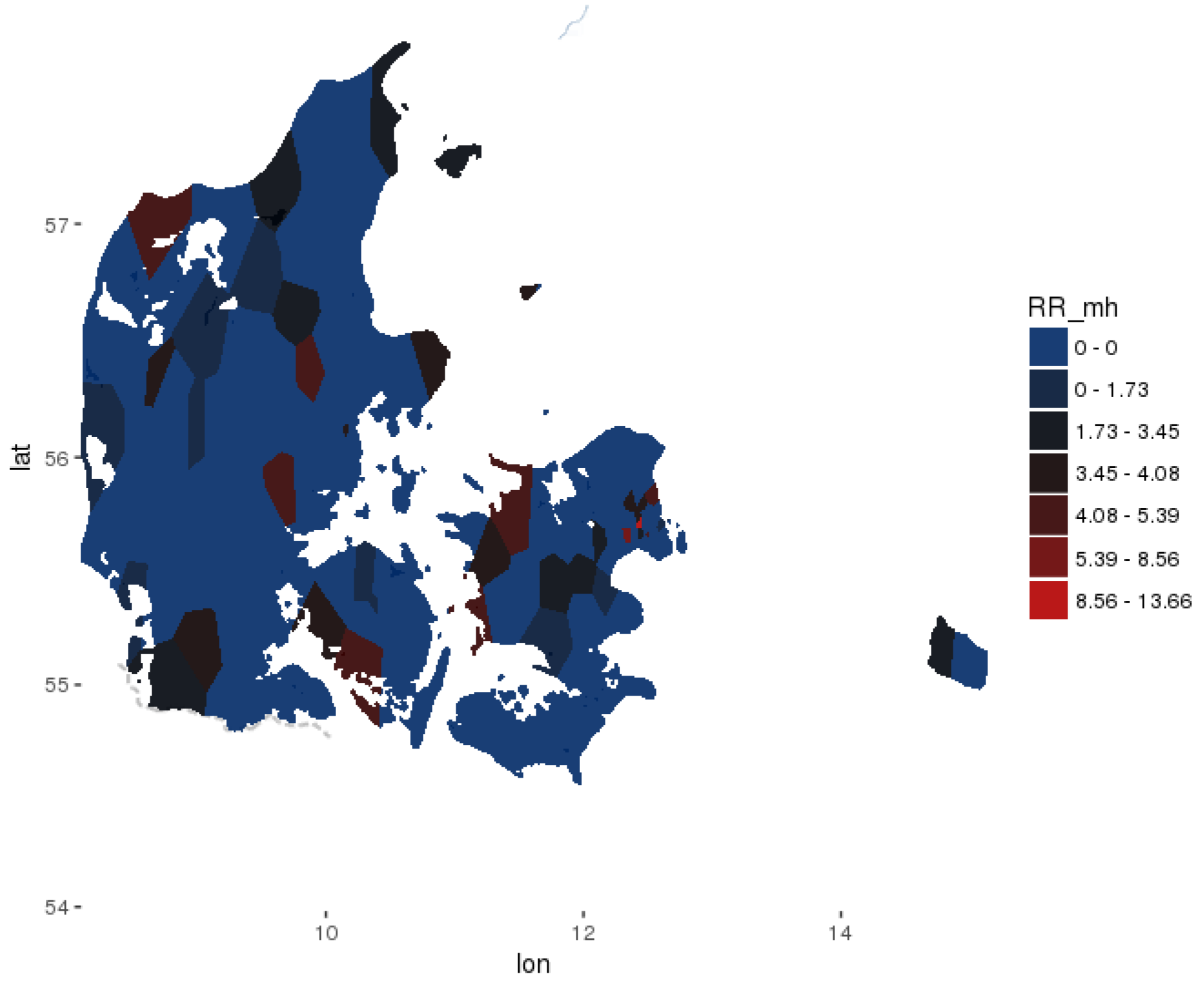

Supplementary Figure 5: Spatial pattern of maternal respiratory infection during pregnancy. The locales were defined as the main empirical analysis with the same risk ratio estimator.

Supplementary Table 1. Demographic characteristics of case-cohort who passed criteria

	Cohort		Case		p-value[1]
**Total** (N)	21700		2328		
**Schizophrenia** (N)	70		2328		
**Other Psychiatric Dx** (N)*	2146		1787		
**History of Psychiatric Dx prior to Schizophrenia** (%)	77%		89%		0.03
**Gender** male (%)	51%		54%		0·03
**Family History** (Yes %)	0·6%		3·6%		$<2x10^{-16}$
**Age - years**	20·4	(SD: 4.1)	24·8	(SD: 4.1)	$<2x10^{-16}$
**Genetic PC 1**	-0·014	(SD: 1)	0·133	(SD: 1)	$4x10^{-12}$
**Genetic PC 2**	0·004	(SD: 1)	-0·038	(SD: 1)	0·04
**Genetic PC 3**	-0·001	(SD: 1)	0·007	(SD: 1)	0·73
**PRS**	-0·020	(SD: 1)	0·230	(SD: 1)	$<2x10^{-16}$

1. The p values were derived from the unweighted marginal tests. Two sample t-test for the continuous variables and the chi-squares for the categorical variables.

* Psychiatric diagnosis other than schizophrenia (ICD10, diagnostic codes in category F but other than F20)

** Genetic scores, including genetic PC, were normalized and mean centered. Among all calculated genetic PC, only first two were significantly different between cases and cohort members. Hence, we only show first three genetic PC here.
